# Prediction of Plastic Shrinkage Cracking of Supplementary Cementitious Material-Modified Shotcrete Using Rheological and Mechanical Indicators

**DOI:** 10.3390/ma16247645

**Published:** 2023-12-14

**Authors:** Kyong-Ku Yun, Valerii Panov, Seungyeon Han

**Affiliations:** 1 Department of Civil Engineering, Kangwon National University, 1 Gangwondaegil, Chuncheon 24341, Republic of Korea; kkyun@kangwon.ac.kr; 2 KICT (Korea Institute of Civil Engineering and Building Technology), 283 Goyang-daero, Daehwa-dong, Ilsanseo-gu, Goyang-si 10223, Republic of Korea

**Keywords:** plastic shrinkage cracking, rheology, crack resistance, supplementary cementitious materials, high-performance shotcrete

## Abstract

Plastic shrinkage cracking is a complex and multifaceted process that occurs in the period between placement and the final setting. During this period, the mixture is viscoplastic in nature and therefore possesses rheological properties. The investigation of the relationship between rheological behavior and its propensity to undergo cracking during the plastic phase presents an intriguing subject of study. However, many factors influence plastic cracking, and the corresponding interaction of its effects is complex in nature. This study aimed to evaluate the impact of rheological and physicomechanical properties on the occurrence of plastic cracking in high-performance shotcrete containing various supplementary cementitious materials. To achieve this, plastic cracking was evaluated employing the ASTM C 1579 standard and a smart crack viewer FCV-30, and the rheological parameters were controlled using an ICAR rheometer. In addition, a study was conducted to assess the strength development and fresh properties. Further, a relationship was established via statistical evaluation, and the best predicting models were selected. According to the study results, it can be concluded that high-yield stress and low plastic viscosity for colloidal silica mixtures are indicators of plastic cracking resistance owing to improved fresh microstructure and accelerated hydration reaction. However, earlier strength development and the presence of a water-reducing admixture allowed mixtures containing silica fume to achieve crack reduction. A higher indicator of yield stress is an indicator of the capillary pressure development of these mixtures. In addition, a series containing ultrafine fly ash (having high flow resistance and torque viscosity) exhibited a risk of early capillary pressure build-up and a decrease in strength characteristics, which could be stabilized with the addition of colloidal silica. Consequently, the mixture containing both silica fume and colloidal silica exhibited the best performance. Thus, the results indicated that rheological characteristics, compressive strength, and water-reducer content can be used to control the plastic shrinkage cracking of shotcrete.

## 1. Introduction

Among the most important issues affecting concrete durability is the formation of plastic shrinkage cracks. Cracking freshly placed concrete occurs in the early hardening stage owing to a change in moisture content and the subsequent development of tensile stresses [1,2]. A crack is a discontinuity or local destruction of a material as a result of the influence of external factors or processes occurring within the structure. The first of such cracks can occur before the initial setting of the moment when the concrete loses its fluidity but has yet to achieve noticeable tensile deformation stability [3,4,5]. The reasons for the formation of plastic cracks are many, which are most intensely manifested at different stages of concrete solidification [6,7,8].

Concrete from the stage it is placed to its final setting is in a plastic state owing to its viscous, flowing behavior, which contributes to its placement, transportation, and formability. Under the action of gravitational forces, coarse and fine aggregates settle, displacing water from the concrete body and thereby causing bleeding [9,10]. However, shotcrete, resulting from spraying and subsequent contact with the surface, compresses to form a dense and rigid structure, which causes the bleeding formation to subside. Further, adhesive and surface tension forces caused by water evaporation result in the formation of internal pressure in the pores of the mixture [11,12,13,14,15]. Plastic shrinkage occurs to compensate for the loss of the liquid phase. Subsequently, compaction of the concrete structure through settlement and evaporation leads to concrete stiffening. At this stage, the concrete is unable to settle, and the hydration reaction is in a dormant state, resulting in the development of shear stresses that eventually lead to plastic cracking. These processes occur during the period from placement to the initial setting, which is referred to as the plastic or viscous phase. Further development of the hydration effect, that is, the development of elastic and strength properties, can restrain the plastic cracking process [16,17].

Rheological properties of the concrete mixture also prevail at this stage. Silica fume is extensively employed in the production and improvement of the technological properties (pumpability and shootability) of shotcrete. Simultaneously, the high specific surface area of silica fume (indicates its fineness) causes the capillary pressure to increase owing to a decrease in the size of capillary pores and an increase in the number of microspores; consequently, the plastic shrinkage increases [13,18,19], increasing the risk of plastic cracking [20,21,22]. From the rheological perspective, owing to the spherical shape of silica fume particles and the effect on the powder size distribution [23], replacing it results in an increase in the yield stress and a decrease in the plastic viscosity (with a replacement ≤ 10%) [24,25,26,27]. Coupled with silica fume, fly ash and its type, ultrafine fly ash, are commonly used, but their application reduces free water content, thereby increasing the risk of plastic shrinkage cracking [28,29,30]. However, certain types of fly ash with an appropriate replacement rate exert a positive effect on plastic cracking resistance [31,32]. In addition, regarding the rheological behavior of fly ash, it has been found that it depends on the replacement percentage. For instance, Szecsy demonstrated that 10% substitution in concrete increases the yield stress, 10–20% reduces it, and 5% reduces the plastic viscosity [33]. Whereas in another study, it was reported that ultrafine fly ash (at 10–20%) reduces the yield stress, and when replaced between 30 to 50%, vice versa [34]. This trend is also related to the fly ash particle size and the effect on the binder particle size distribution [35]. Recently, it was discovered that colloidal silica can improve the crack resistance of shotcrete mixtures [36], owing to the early strength development [36] and the formation of a flocculent layer capable of retaining free water [37,38]. Furthermore, nano-silica particles have been found to reduce the fluidity of mixtures. Kolawole pointed out a correlation between the yield stress and thixotropy index with the occurrence of plastic shrinkage and capillary pressure development in his study [39]. Thus, based on the brief review above, a relationship between the characteristics of plastic cracking and rheological parameters can be concluded.

During the plastic stage (before initial setting), evaporation, settlement, shrinkage, and the formation of capillary pressure are prevailing processes, affecting the onset and extent of cracking. The subsequent development of the hydration effect (after the initial setting time), which develops the concrete strength, can help to withstand plastic shrinkage stresses and plastic cracking [40]. The period between the initial and final setting is defined as the semi-plastic phase, wherein the plastic cracking phenomenon is greatly influenced by the tensile strength and tensile strain capacity, as well as the stiffness and relaxation of concrete [41,42]. In other words, early strength development can serve as an indicator of resistance to tensile stress causing cracking, and acceleration of the hydration reaction can lead to early setting, thereby reducing the time of exposure to the negative effects of climate conditions. Thus, the development of mechanical characteristics can prevent or delay the occurrence of plastic shrinkage cracking. In addition, both phases of the concrete’s hydration process (plastic and semi-plastic) must be considered to assess plastic cracking.

This study presents an assessment of the plastic shrinkage crack resistance of high-performance shotcrete with the addition of various supplementary cementitious materials, in particular, silica fume, fly ash, and colloidal silica of various particle dimensions. Using the method described in ASTM C 1579 [43] and a smart crack viewer (FCV-30) [44], the average crack widths were measured. Consequently, the values obtained were compared with the physicomechanical and rheological characteristics of the shotcrete, such as strength and fresh state characteristics, with the aid of statistical analyses. As a result, the factors influencing plastic cracking were determined for diverse supplementary cementitious materials. To this end, the experimental campaign was divided into several sections. Section 2 is devoted to the mix design development, selection of materials, and a detailed procedure for evaluating the shotcrete behavior. Section 3 discusses the following aspects: cone slump and air content in fresh concrete; plastic shrinkage cracking; strength characteristics; rheological behavior; and the relationship of all variables using statistical methods. Subsequently, Section 4 presents an analysis of the results and conclusions, comparing them with previous studies. Finally, Section 5 presents the conclusion of the study and its significance and limitations.

## 2. Materials and Experimental Methods

### 2.1. Materials

**Figure 1:**
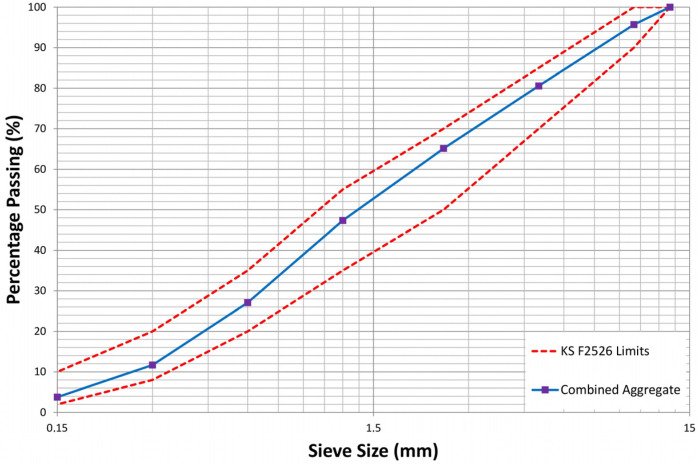
Particle size distribution curve of combined aggregates used for shotcrete mixture production.

River sand and crushed stone were employed as coarse and fine aggregates, respectively, in a 75% sand/aggregate ratio (S/a), satisfying the Korean quality standards [45,46]. The coarse aggregate has a maximum grain size of 10 mm (G_max_), a specific gravity of 2.69, and an absorption of 1.95%. In contrast, fine aggregate has a specific gravity of 2.62 and an absorption of 0.61%. Figure 1 illustrates the particle size distribution curve of the combined aggregates employed in this study.

The primary binder employed in this study was ordinary Portland cement type I (OPC) produced by Sungshin Cement Company, Seoul, Republic of Korea, and complying with the KS L 5201 standard [47]. Further, to study the changes in plastic behavior, the following variables in mixtures were considered: a 40% solution of colloidal silica (CS) with a particle size of 10 nm, 40 nm, and 80 nm manufactured by Youngil Chemical. Co. Ltd., Incheon, Republic of Korea; adding silica fume (SF); and a combined application of colloidal silica with ultrafine fly ash (UFFA) and silica fume produced by Chemius Korea Company, Seongnam, Republic of Korea. In addition, to maintain the required flowability and plasticity (120 ± 20 mm slump according to standard recommendation for shotcrete [46]), the water-reducing agent (AEWR), which included 0.2% of the air-entraining agent, manufactured by Silkroad C&T Co. Ltd., Seoul, Republic of Korea, was dosed individually for each mixture. Physicochemical indicators of these cementitious materials and admixtures are shown in Table 1.

### 2.2. Concrete Mixture Compositions

For the experiment, 10 mixtures were developed using various supplementary binders. However, the volume of cementitious materials and W/B remained constant at 460 kg/m^3^ and 40%, according to the Korean standard recommendation [46], respectively. Further, colloidal silica of 10 nm particle size was used at 2, 3, and 4% volume percentages. Whereas, for mixtures with 40 and 80 nm-sized particles, the percentage of colloidal silica used was 3% to study the effect of particle size change. Further, 4 and 7% substitution values of silica fume were used. The mixtures denoted as SF4.CS2.10 and FA20.CS2.10 were developed to determine the behavior of the combined application of supplementary cementitious materials. These mixtures included 20 and 4% of ultrafine fly ash and silica fume, respectively. The resulting mixture combinations are shown in Table 2.

In addition, Table 3 provides a summary of the factors (percentage of colloidal silica, particle size of colloidal silica, percentage of silica fume, and percentage of ultrafine fly ash) and their corresponding levels for each experimental run in the mixture composition. Each row represents a unique combination of factors, facilitating a systematic investigation of their effects on mixture properties such as cone slump and air content in fresh concrete, plastic shrinkage cracking, strength characteristics, and rheological behavior. Moreover, the inclusion of AEWR admixture was considered a factor affecting the resulting plastic shrinkage cracking properties.

### 2.3. Experimental Test Methods

#### 2.3.1. Air Content and Slump Measurements

The standard cone was used to evaluate the workability of the shotcrete mixture according to the standard KS F 2402 [48], and the pressure method was employed to control entrained air before and after spraying [49].

#### 2.3.2. Compressive and Flexural Strength Tests

Compressive and flexural strength tests were conducted on cylindrical specimens with dimensions of 100 × 200 mm (three specimens) and beam specimens of 100 × 100 × 400 mm (two specimens) maintained at a temperature of 25 °C and an RH of 50% for 24 h within a special room. Subsequently, they were transferred to a chamber that maintained normal hardening conditions (20 °C and 80% relative humidity). Thereafter, the specimens were tested using a universal testing machine using controlled loading conditions of 0.6 ± 0.4 MPa/s for the compressive strength test and 0.06 ± 0.04 MPa/s for the flexural strength test. These tests were conducted in accordance with the relevant Korean standards [50,51]. The flexural strength and ultimate compressive strength of the specimens were determined using Equations (1) and (2), respectively.

(1) $$f_{b}=\frac{Pl}{bh^{2}}$$

(2) $$f_{c}=\frac{P}{\pi {\left(\frac{d}{2}\right)}^{2}}$$

where *f_b_*—flexural strength (MPa), *f_c_*—compressive strength (MPa), *P*—maximum obtained load (N), *l*—span length (mm), *b*—width of the failed cross-section (mm), *h*—height of the failed cross-section (mm), and *d*—diameter of specimen (mm).

#### 2.3.3. Plastic Shrinkage Cracking Detection

**Figure 2:**
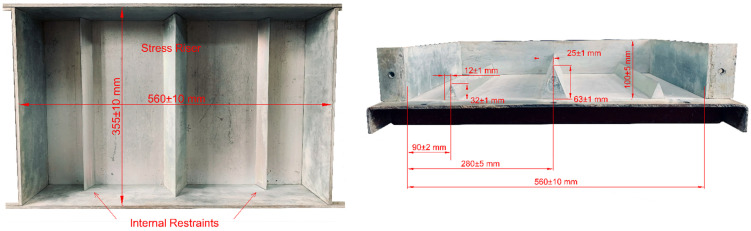
Test plate configuration according to ASTM C 1579.

**Figure 3:**
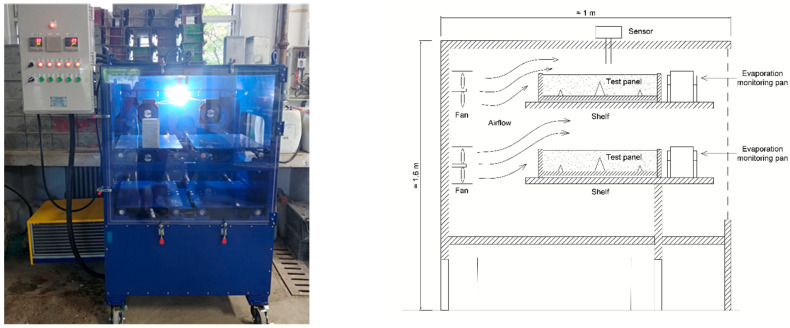
Climate control chamber configuration for creating conditions causing shrinkage cracking.

**Figure 4:**
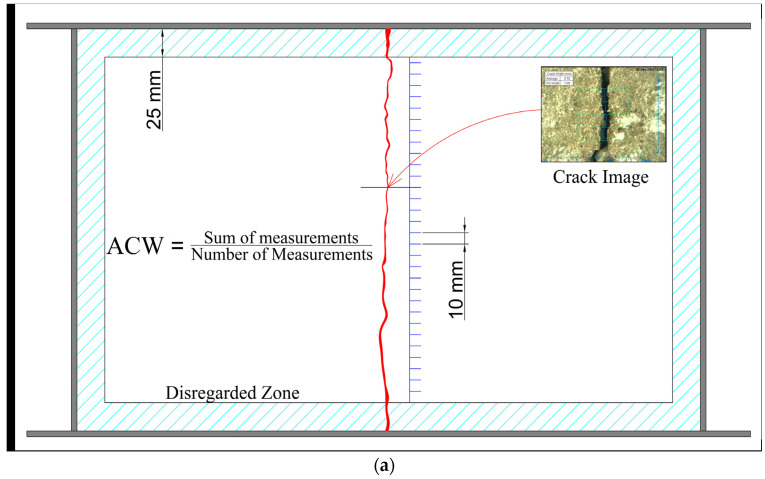

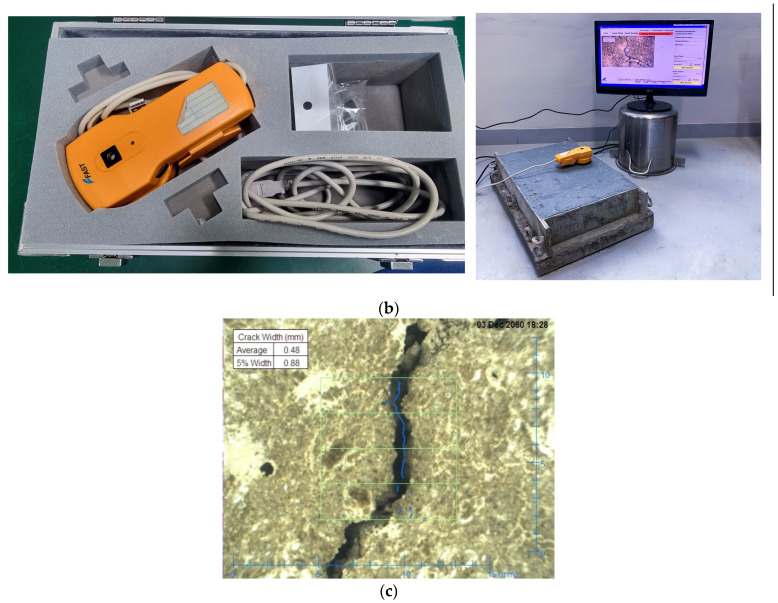
Cracking evaluation. (**a**) crack measuring procedure, (**b**) crack viewer FCV-30, and (**c**) image data after measurement.

The method for assessing plastic shrinkage cracking that was employed in this study is explained in ASTM C 1579 [43]. The purpose of this test method is to evaluate the impact of evaporation, settlement, and early shrinkage on the properties of plastic cracking during the plastic and semi-plastic stages of concrete. That is, the evaluation can be conducted before and within several hours after the concrete’s final setting. The measuring procedure consists of the following stages: placing concrete in a test plate, exposing the plate to extreme climate conditions generating excessive evaporation, and the final measurement of the average crack width. The laboratory equipment used included a test plate, a climate chamber, and a crack viewer FCV-30 [44], as shown in Figure 2, Figure 3 and Figure 4. Further, the test mold used in the study was composed of a metal having a depth of 100 ± 5 mm, a width of 355 ± 10 mm, and a length of 560 ± 10 mm. In addition, two smaller metal inserts (32 ± 1 mm) on the sides acted as internal restraints. A large metal insert measuring 63 ± 1 mm in the middle was used as a stress riser, causing the cracking. Thus, the configuration used allowed cracks to form in the middle of the test mold. Furthermore, to prevent the concrete from sticking to the mold, all test plates were oiled prior to testing.

The climatic chamber was maintained at a temperature of 36 ± 3 °C after the start of the test and a relative humidity of 30 ± 10%, which was controlled using a monitoring pan after reaching 28% RH. Further, a fan that generated a wind speed of 4.7 m/s to maintain an evaporation rate of 1.0 kg/m^2^ h was installed. These parameters were maintained continuously for all test series to create optimal crack formation conditions.

After a period of creating optimal climatic conditions (24 h), the average crack width of the samples was measured. The crack width was estimated at 10 mm intervals, and any crack in the 25 mm range around the mold was disregarded, as shown in Figure 4a. The crack reduction ratio (*CRR*), expressed in Equation (3) [43], was used to evaluate the effects of various supplementary cementitious materials. The method of comparison of the control sample with the test sample was applied; for instance, a sample OPC with CS2.10. The trial units consisted of at least two control specimens (each time) and at least two test samples with the same mixture composition [43].

(3) $$CRR=\left[1-\frac{Average\;Crack\;Width\;of\;the\;Test\;Concrete\;Mixture}{Average\;Crack\;Width\;of\;Control\;Concrete\;Mixture}\;\right]\times 100$$

Subsequently, the final cracks were evaluated using the intelligent crack viewer FCV-30 [44] by analyzing the sample images. This crack viewer (Figure 4b) is equipped with a 1.4-million-pixel camera for assessing cracks in 0.01 mm increments and can measure crack widths up to 2.0 mm. Crack detection is realized via image processing, wherein cracks are typically identified using edge elements, as the edges can indicate a crack. The cracks are represented as abrupt changes in grayscale values between edges and adjacent pixels, forming an outline and appearing in images as blue lines (Figure 4c). The viewer software processes the image, and all crack widths recognized in the 8 × 8 mm image range (green mesh) are recorded. Consequently, the program displays a histogram of crack width distribution and calculates the average crack width. FCV-30 is capable of measuring the crack width with an accuracy of ±0.02 mm.

#### 2.3.4. Rheological Measurements 

**Figure 5:**
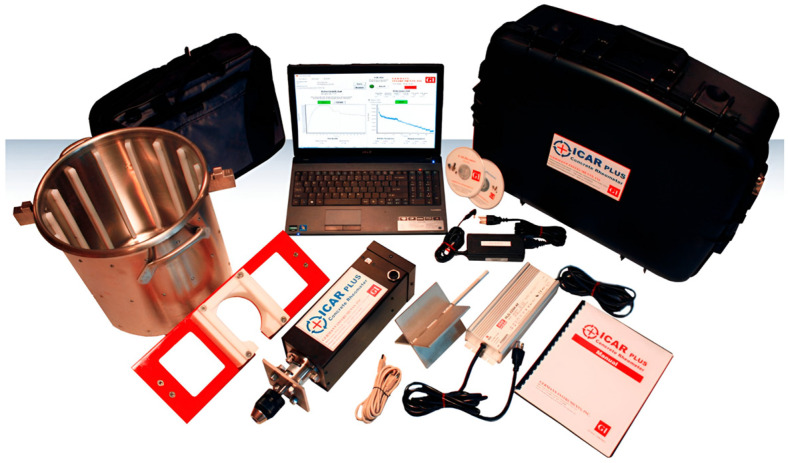
ICAR Rheometer.

Fresh concrete flows and behaves similarly to liquid material until it hardens owing to hydration [52]. The field of science that studies the mechanics of such a flow and its deformation is referred to as rheology. The relationship between shear rate and shear stress can be used to characterize the viscous flow of concrete mixtures [53]. The constant used within this ratio is referred to as the plastic viscosity, while the transformation stress that initiates the transition from the liquid-state to the solid-state behavior is referred to as the dynamic yield stress [25]. These parameters can be obtained using the flow curve test employing an ICAR rheometer [54] (Figure 5). Consequently, based on the measurement of the average torques and rotation speeds, a regression curve is plotted, wherein the point of intersection of the curve with the y-axis represents the flow resistance (*G*) and the slope of the curve represents the torque viscosity (*H*). Using the Bingham model, the ICAR Plus software (version: 3.0.0.82) can convert the *G* and *H* values into the fundamental values of dynamic yield stress and plastic viscosity. However, the measured shear speed is proportional to the converted shear rate, and the measured torque is proportional to the shear stress obtained for fundamental properties [52,53]. Therefore, in the framework of the present study, non-fundamental parameters (*G* and *H*) are used. These values are sufficient for studying the rheological behavior (trends) and explaining the effect of admixtures.

#### 2.3.5. Statistical Analysis

A statistical study was conducted to compare the effects of different physical properties of shotcrete on plastic cracking. The correlation analysis was performed to identify the links between cracking properties and rheological constants with mechanical properties using the Pearson correlation coefficient. Further, correlated variables were selected to determine the correct model for predicting plastic cracking resistance by using simple and multiple regression models. For each regression model, the optimal fitting equations were estimated to explain the behavior of the dependent variable, considering the regression statistics (coefficients of determination *R*^2^ and *R*^2^*adj*) and the penalized-likelihood criteria Akaike (*AIC*) and Bayesian (*BIC*). Furthermore, the *p*-value was used to assess the significance level of all variables, and 95% confidence intervals were determined for additional control of each simple regression model [55].

## 3. Experimental Test Results

### 3.1. Slump and Air Content Tests

In this study, the cone slump was maintained at 120 ± 30 mm, while the entrained air content was controlled within the limit of 7 ± 2% to ensure similar plastic conditions for all test series by using AEWR. AEWR possesses the ability to reduce capillary pressure in the meniscus by reducing the rate of evaporation [56]; therefore, the indicator of its content can be used to characterize the ability of a mixture to resist cracks in the plastic phase. Another important indicator for shotcrete is the air content after spraying.

**Figure 6:**
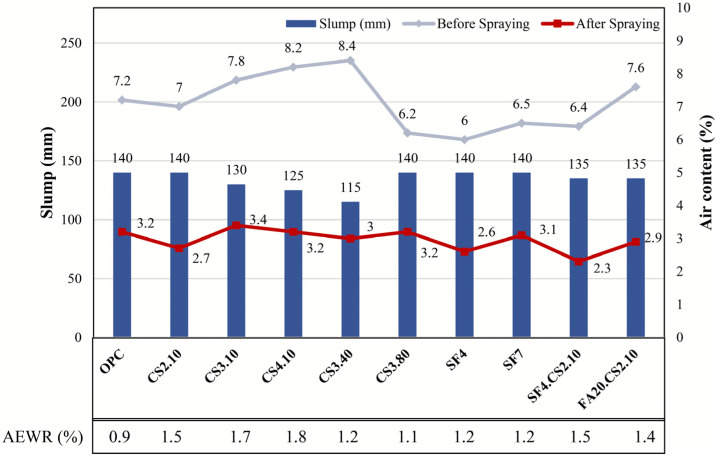
AEWR content, resulting slump, and air content for different mixture combinations.

As indicated by the results of the previous study [36] and confirmed by current research, an increase in the percentage of CS replacement results in a reduction in the flowability of the mixture (slump) and an increase in the air content before the start of the shotcreting process. Thus, this situation requires an increase in the AEWR content along with that in the CS content. Further, although mixtures with the combined application of UFFA with CS and SF with CS demonstrated similar behavior in terms of workability, the series with the inclusion of FA exhibited an increase in the air content before spraying in the mixture (compared with SF4.CS2.10). Moreover, the series with 40 and 80 nm particle sizes had less effect on workability than the 10 nm CS (reduction in AEWR). Simultaneously, an increase in stiffness relative to OPC was observed. SF4 and SF7 had the same effect on the workability of the mix, and less AEWR was used to ensure their flowability than for the CS series. In addition, the air content after spraying varied from 2.7 to 3.2%, which varies depending on the skill of the operator performing the spraying and consolidating process [57]. Thus, as the shotcrete process has an air-reducing feature, a decrease in the entrained air by 4–5% was observed in each series. The above results are shown in Figure 6.

### 3.2. Plastic Shrinkage Cracking Evaluation

**Figure 7:**
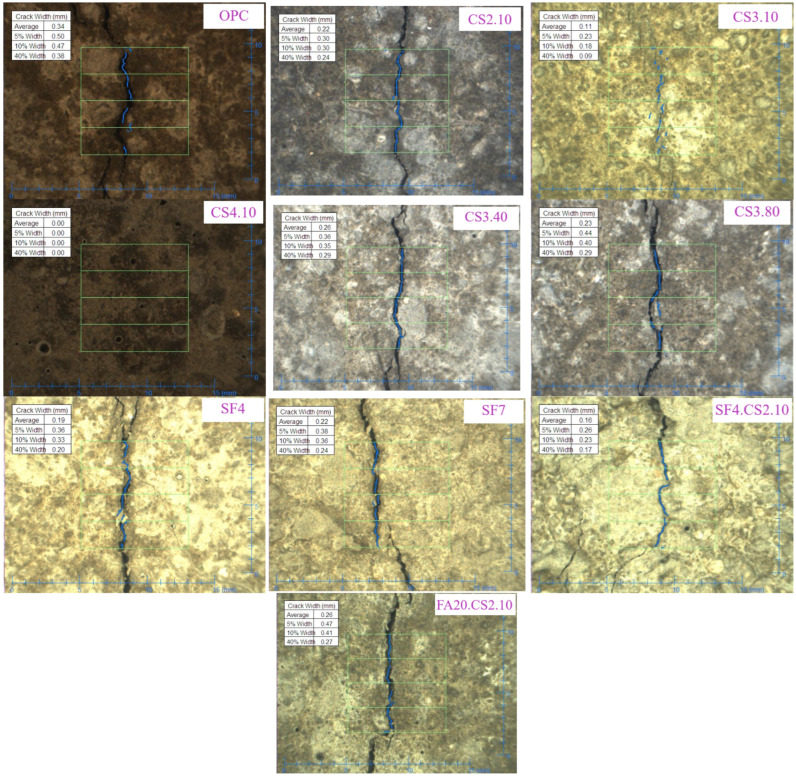
Obtained image examples of shotcrete specimens.

For each series, two test specimens were cast, and the comparison was performed using the *CRR* coefficient expressed in Equation (3). Further, the images obtained via the crack viewer (Figure 7) were used to analyze the average crack widths using the software. In addition to the average width, the program calculated 5, 10, and 40% deviations from the average width. However, previous research [36] has shown a similar trend in these values; thus, the analysis was conducted based only on the average widths. 

#### 3.2.1. Plastic Shrinkage Cracking Behavior of CS Mixtures 

**Figure 8:**
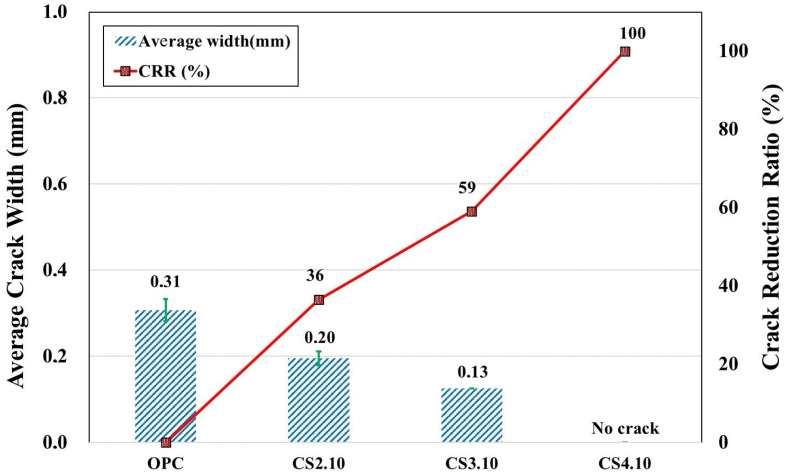
Resulting average crack widths and *CRR* coefficients of colloidal silica specimens.

An experimental study has shown that colloidal silica with a 10 nm particle dimension significantly improves the crack resistance of the shotcrete mixture when its replacement percentage is increased. This behavior is demonstrated in the example shown in Figure 7. Since this admixture forms a flocculent layer capable of retaining free water [37], this effect is observed at an early plastic stage. Consequently, the rate of water evaporation from the surface of fresh concrete decreases, resulting in a decrease in the rate of capillary pressure. Therefore, the average crack width decreases with an increase in the CS percentage from 2 to 4%. However, in the case of the CS10.4 mixture, the crack did not occur, as shown in Figure 8. The observed behavior can be attributed to a combination of factors: the advancement of significant early strength, as demonstrated in Section 3.3, which enables it to endure tensile stress; the impact of the CS itself; and the influence of the AEWR addition. Consequently, this enhances the ability to withstand early plastic shrinkage cracking, preventing the formation of cracks. Thus, based on the results of the CRR assessment, it is evident that CS of 2 and 3% reduces the plastic shrinkage crack by 36 and 59%, respectively, while at 4%, no crack is formed. This indicates that the CS replacement can improve the plastic shrinkage crack resistance of high-performance shotcrete to a certain extent and is consistent with the results of previous research [36].

#### 3.2.2. Plastic Shrinkage Cracking Behavior of CS Mixtures with Different Particle Sizes 

**Figure 9:**
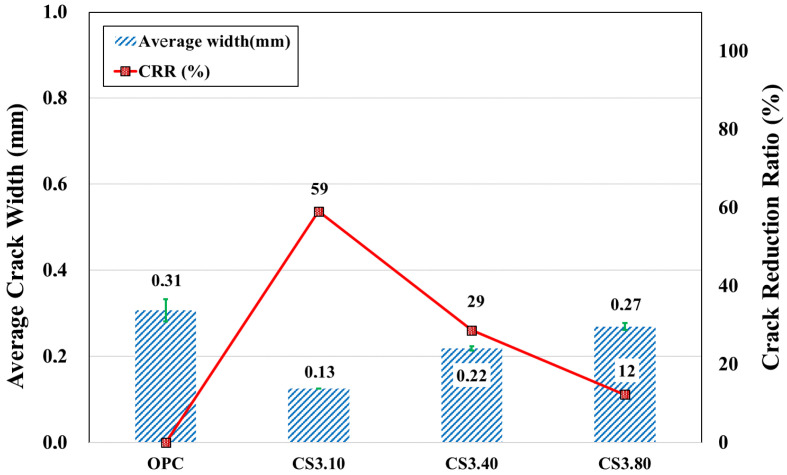
Average values of plastic shrinkage cracking and *CRR* indicators of colloidal silica specimens of various particle dimensions.

Figure 9 shows the average width of plastic cracking for the CS series with different particle sizes. According to the graph, the crack widths for 40 and 80 nm decreased compared to OPC. However, a mixture with 10 nm-sized particles exhibited a greater reduction. After analyzing the *CRR* for mixtures CS3.40 and CS3.80, it was found that the crack size decreased by 29 and 12%, respectively, indicating that increasing the particle size of the colloidal silica results in a decrease in the influence of colloidal silica on crack resistance. Consequently, the resistance to plastic cracking deteriorates. One possible reason may be the lower amount of AEWR in mixtures with the addition of 40 and 80 nm CS. As the content of the water reducer decreases, its positive effect on evaporation decreases as well. Therefore, the optimal CS size was concluded to be 10 nm, but 40 and 80 nm (to a lesser extent) may also be used considering environmental conditions.

#### 3.2.3. Plastic Shrinkage Cracking Properties of SF Mixtures

**Figure 10:**
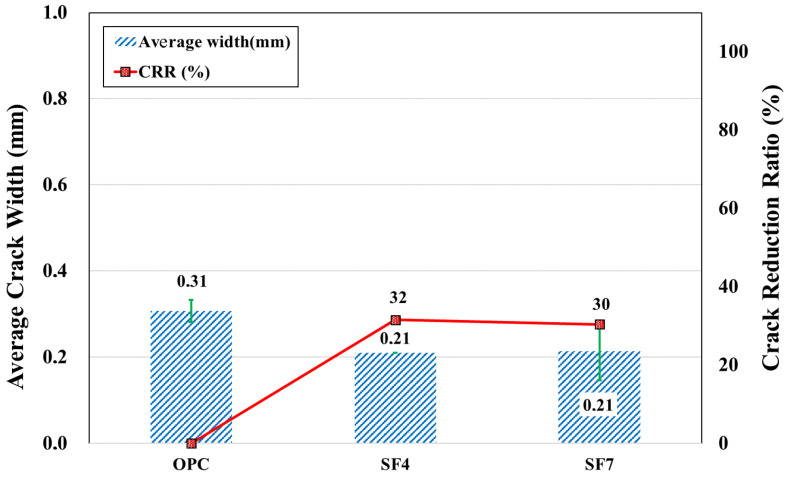
Average crack width and *CRR* of silica fume samples.

The SF admixture is frequently used to produce high-performance shotcrete and improve shootability [58]. However, the high capacity to absorb free water and reduce capillary pore size results in negative consequences; this tendency leads to an increase in capillary pressure, which consequently increases the risk of plastic shrinkage cracks [13]. However, the opposite behavior occurred here. The measurement conducted for the plastic shrinkage cracking at the age of 24 h yielded a 0.21 mm crack for the mixtures SF4 and SF7, as shown in Figure 10. This is less than that observed in the control OPC specimen with a 0.31 mm-average crack width, as in Figure 6. Further, according to the *CRR* coefficient, SF reduced the width of plastic cracks by 28%. Such a kind of behavior is possible, and certain studies have exhibited these results [59]. 

#### 3.2.4. Characteristics of Mixtures Crack Resistance with a Combination of SCMs

**Figure 11:**
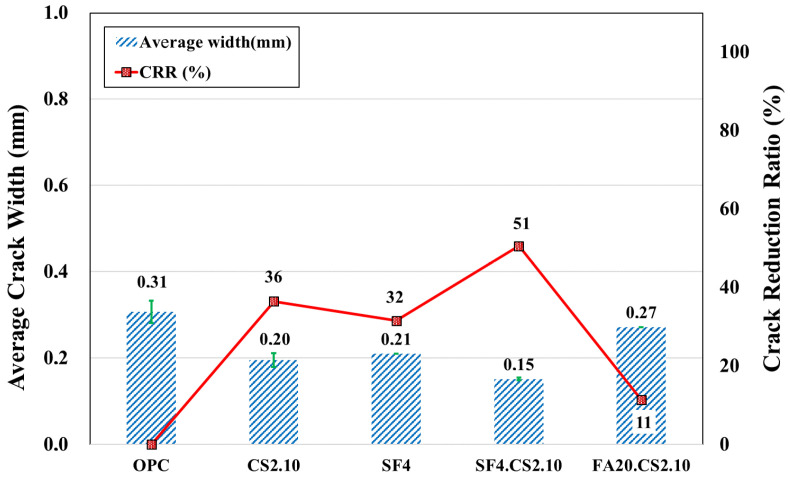
Average width of plastic shrinkage cracks and *CRR* of mixtures with a combination of SCMs.

The mixtures SF4.CS2.10 and FA20.CS2.10 were used to demonstrate the complex effect of various admixtures on plastic shrinkage cracking in this study. As stated earlier, silica fume exhibited increased crack resistance despite its ability to absorb free water. However, the combination of 2% CS and 4% SF showed a greater effect on the average crack width of the specimen. However, if, with their separate applications, 0.20 mm (CS2.10) and 0.21 mm (SF4) cracks were formed, then the combined replacement decreased to a value of 0.15 mm. Based on the *CRR* analysis, the average crack width decreased by 48%. Moreover, this value was significantly higher than that for SF4 and CS2.10, with a reduction of 36% and 32%, respectively, indicating that the use of SF and CS reduces the risk of cracking more efficiently than a separate application. Further, the series containing 20% ultrafine fly ash exhibited no significant reductions, whereas CS2.10 showed better performance. This indicates that when FA is used, the effect of CS on plastic cracks is reduced. However, fly ash mixtures are highly susceptible to plastic cracking owing to their high water absorption capacity [29]. Therefore, it can be concluded that CS stabilizes FA, and their combined application can counteract this defect. These results are shown in Figure 11.

### 3.3. Strength of Shotcrete Material

Drying and an increase in capillary pressure result in plastic shrinkage. However, the extent of plastic shrinkage cracking is not proportional to capillary pressure and plastic shrinkage. For sufficient concrete ultimate tensile strength, the effects of such factors are minimized, which can be achieved by accelerating the hydration process (subsequent early strength development) caused by the addition of diverse supplementary cementitious materials [60]. Consequently, plastic cracking does not occur or forms with a smaller width.

**Figure 12:**
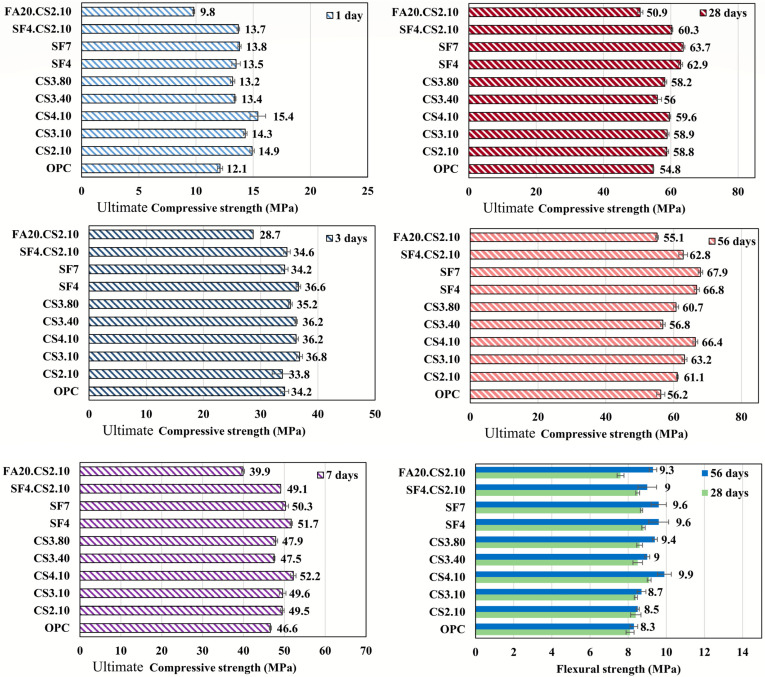
Compressive and flexural strength development of diverse shotcrete mixtures.

As previously demonstrated [36], ultimate compressive strength is an important factor that can be used to assess plastic shrinkage crack resistance. Figure 12 summarizes the ultimate compressive strength of the material at a daily age, and the influence of supplementary cementitious materials on the hydration reaction was evaluated by considering strength parameters at various design ages. The ultimate strength of the material containing CS was found to have increased with their content, owing to the filler ability of the admixture to form a denser microstructure and high-strength indicators due to the pozzolanic reaction [38]. This effect correlates well with the results obtained from the measurement of plastic cracking. The changes observed in compressive strength from the first day up to 28 days later indicated that hydration experienced an acceleration caused by the addition of colloidal silica [61,62]. Consequently, a final setting time was approached, thereby reducing the period of exposure to the environment on fresh concrete. Further, changing the CS particle size from 10 nm to 40 and 80 nm reduced the strength characteristics of the mixture, demonstrating a lower crack resistance than that of CS3.10. In addition, the ultimate strength was developed to a lesser extent at different ages of hardening, which may be because larger particles encounter difficulties in dispersing within the mixture. Moreover, mixtures with 4 and 7% replacement of SF exhibited strength development at an earlier stage owing to hydration reaction acceleration [63,64], and the compressive strength of these mixtures was greater than that of conventional shotcrete. The earlier development of the parameter of SF mixtures resulted in a reduction in the average crack width. In addition, SF4.CS2.10 exhibited a strength similar to the SF series, although the crack resistance was at a level higher than that observed in the separate applications of CS and SF. It can be concluded that the effect of CS, which consists of retaining free water, was improved with the combined use of these admixtures. The shotcrete containing FA showed the worst compressive strength compared to the rest of the series because fly ash reduced the heat of hydration (hindering the hydration process), thereby reducing the resulting mechanical performance [65]. However, the average crack width was lower by 5% than that in the OPC. Therefore, in combination with CS, its effect can aid in restraining the plastic cracking of mixtures containing FA. Furthermore, all the series of specimens achieved a typical value of compressive strength (28 MPa) at the age of 28 days [46].

Because under the influence of a flexural load, tensile stresses are formed at the edge of the specimen, which destroys the beam sample. Therefore, a relationship between cracking and flexural strength is an interesting prospect. CS contributes to an improvement in flexural strength upon reaching 28 and 56 days. Moreover, an increase in the dimension of its particles results in increased resistance to flexural stress. Further, SF mixtures also produce a structure with increased strength, and consequently, the combination of SF and CS demonstrates its effectiveness in this regard. In addition, the mixture FA20.CS2.10 with a flexural strength less than the standard [46] one (8 MPa at 28 days) showed strong development at the age of 56 days. 

### 3.4. Rheological Properties

Based on the measured torque and rotational speed data, a regression curve (Bingham model) was drawn for each test series, and the curve parameters are presented in Table 4. The CS mixture in a fresh state showed an increase in yield stress and a decrease in torque viscosity in proportion to the percentage of CS inclusion. Further, a change in the particle size of the CS caused reduced yield stress and plastic viscosity compared to those for the 10 nm particle size. The results are consistent with those obtained in another study [66]. In addition, as indicated earlier [26,58], SF increases the flow resistance of shotcrete and decreases the torque viscosity value. Moreover, the combination of SF and CS showed an even greater influence on yield point and plastic viscosity than those exhibited by the mixtures of SF4 and CS2.10. The mixture FA20.CS2.10 had the highest flow resistance and a high torque viscosity. The values obtained were analyzed using statistical tests to understand the factors that influenced these results.

### 3.5. Statistical Analysis

#### 3.5.1. Analysis and Study of Factors Influencing Plastic Shrinkage Cracking

**Figure 13:**
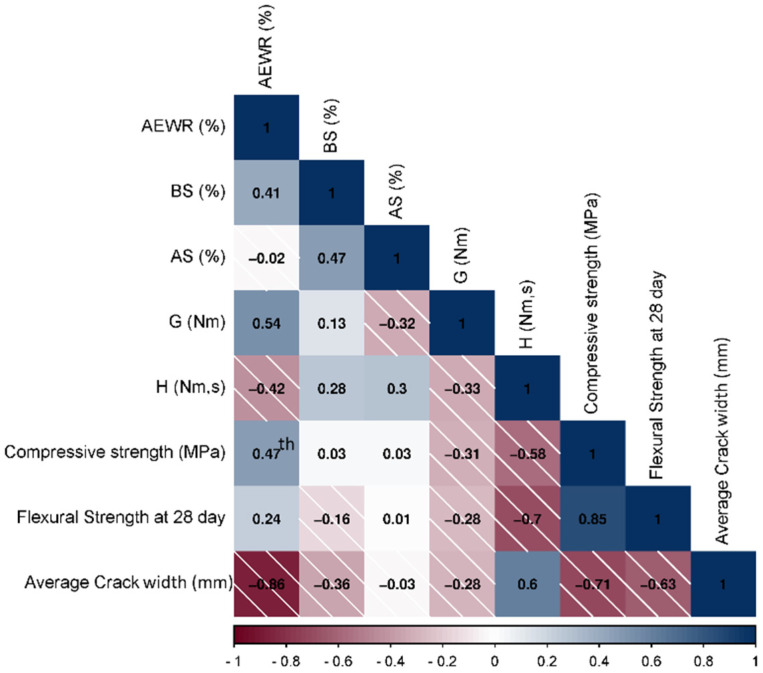
Pearson correlation coefficients for all experimentally determined factors and mixtures.

To analyze the crack resistance and the factors affecting it, all the variables obtained and discussed in the previous sections were divided into subgroups, as shown in Table 5. The groups included the characteristics of the mixture composition, that is, the content of AEWR. However, other variables of the mixture, such as the supplementary cementitious material replacement, were not included as they were estimated using the *CRR*. In addition, the air content in the fresh mixture (before and after spraying) and the rheological parameters (flow resistance and torque viscosity) were considered indicators that characterized the plastic phase. Further, strength features such as compressive (24 h) and flexural (28 days) strengths were used to characterize the semi-plastic and solid phases. The links and the relationship between these variables were assessed using the Pearson correlation coefficient and its corresponding two-tailed test. The results are summarized in Figure 13.

From Figure 13, it is evident that a low correlation exists between entrained air content before spraying (BS) and crack width (*r* = −0.357), which is consistent with the results of a study that reported that only high air content has an impact on the risk of cracking [67,68]. Further, the air content after spraying (AS) showed no relationship (*r* = −0.031); however, this indicator can be related to the yield stress obtained after spraying [69], which ultimately affects the mixture stiffness. The AEWR factor shows a strong negative correlation with the crack width (*r* = −0.863) at 1% significance, owing to the capillary pressure-reducing properties of AEWR [56]. Moreover, the compressive strength exhibited a high negative correlation (*r* = −0.711) at a significance level of 5%, which is consistent with Section 3.3. In addition, an average negative correlation was also observed for flexural strength (*r* = −0.628), but it is not significant, probably because the flexural strength was measured on the 28 days where cracks do not propagate. However, this factor is related to the compressive strength (*r* = 0.847), and both of these indicators characterize the strength development, although the compressive strength is considered more optimal. The torque viscosity also showed a substantial relationship (0.604), which indicates the possibility of a relationship but is not significant for *H*, and the yield stress is not correlated. Further, the moderate relationship between the AEWR and *G*, *H*, and compressive strength is also visible. Another interesting observation is the significant relationship between plastic viscosity and flexural strength (*r* = −0.697 at a significance level of 5%). Owing to the fact that different compositions produce different effects on plastic stage properties, it is necessary to consider several models explaining materials with similar behavior. To consider this behavior of mixtures modified with various cementitious materials in detail, certain observations needed to be excluded to find the optimal regression model for characterizing plastic cracking. Consequently, three main models can be distinguished based on the results of the correlation analysis:

- Model I includes all observations, compressive strength, and AEWR content variables.
- Model II eliminates silica fume mixtures (compressive strength, AEWR content, and plastic viscosity predictors).
- Model III was based only on mixtures with colloidal silica application (Model II variables and flow resistance).

**Figure 14:**
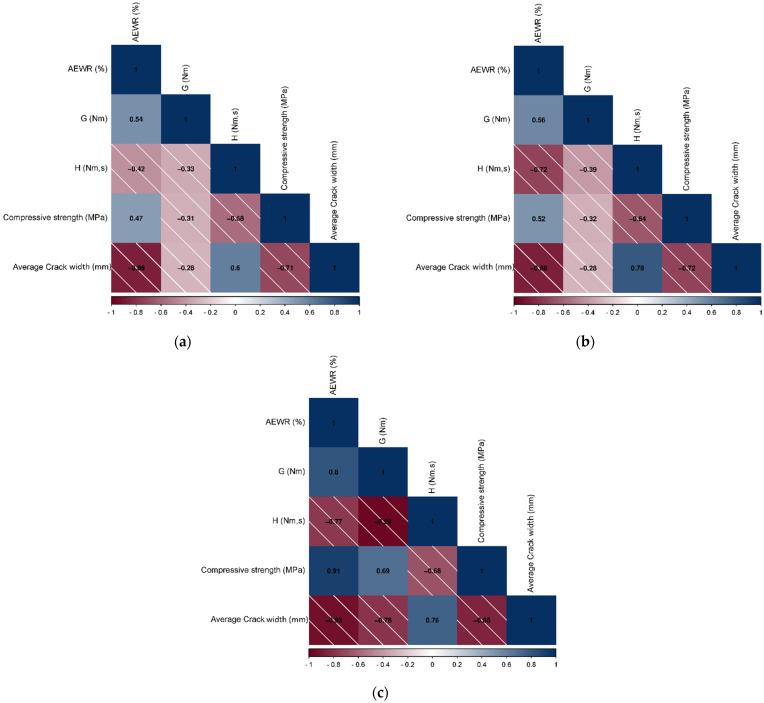
Pearson correlation coefficients for explanatory variables used. (**a**) Model I, (**b**) Model II, and (**c**) Model III.

For Model I, AEWR and compressive strength yield high values of the Pearson correlation (Figure 14a). Thereafter, using these indicators, a multiple regression model was compiled and analyzed (Table 6), which showed significance at the 0.1% level along with a high adjusted determination coefficient (*R*^2^*adj* = 0.824). All variables included in this model are significant (*p*-value < 0.05), indicating that this model can explain the change in plastic cracking of all mixtures by 86%.

Upon analyzing Model II, the Pearson correlation coefficients for the compressive strength and water-reducer content were found to have increased (Figure 14b). Moreover, plastic viscosity and plastic shrinkage cracking showed a significantly positive relationship (*r* = 0.783). However, the AEWR content and rheological indicators (*G* and *H*) exhibited a correlation as well. This is because fly ash and colloidal silica reduce the workability of the mixture, and their introduction causes the required amount of water reducer to increase, as shown in Section 3.1. Moreover, a moderate correlation can also be observed between the compressive strength and AEWR because AEWR improves the performance of the concrete by reducing the required amount of water in the mixtures [70]. The results of the multiple regression analysis tests indicate that this predictive model has high regression statistics indicators (*R*^2^ = 0.877 and *R*^2^*adj* = 0.785) and is significant at the 0.027 level. However, each predictor is not as significant individually because of the correlation of these variables (multicollinearity). This case is an indication that plastic cracking behavior is independent of each factor separately but depends on the complex influence of groups of indicators. Furthermore, this model can describe the variables to a certain extent; that is, the cracking resistance of samples containing colloidal silica and ultrafine fly ash is dependent on the water reducer content, strength characteristics at an early stage, and plastic viscosity value.

If a mixture containing fly ash is excluded, an interesting result in the indicator correlation is observed (Figure 14c). A possible strong negative correlation (*r* = −0.773) between *H* and AEWR exists because the water reducer reduces the zeta potential [71] and thereby decreases viscosity and yield stress, as reported in other studies [72,73]. Further, it may also be because of an increase in the colloidal silica content owing to the increase in yield stress, and a higher concentration of this admixture is required (*r* = 0.802). The very high negative correlation between *H* and *G* (r = −0.977) supports this argument, i.e., the colloidal silica influence on concrete rheology serves as an explanation of such behavior. In addition to the already-found correlations with AEWR, strength, and viscosity, a strong negative correlation was also observed between yield stress and cracking (*r* = −0.785). Further, the use of multiple regression for Model III with the inclusion of all explanatory variations resulted in the observation of a weak significance level (more than 95%), which was because of the high multicollinearity. Therefore, Model III was broken down into sub-models with a combination of various indicators. Table 6 shows that all paired indicators have high determination coefficients (*R*^2^ = 0.785–0.870) and good-adjusted determination coefficients (*R*^2^*adj* = 0.677–0.805), and the models are significant. Therefore, it can be concluded that the stability of mixtures containing colloidal silica to plastic cracking is influenced by all predictors, and the influence is expressed in their complex interactions.

#### 3.5.2. Simple Regression of Plastic Shrinkage Cracking Predictors

**Figure 15:**
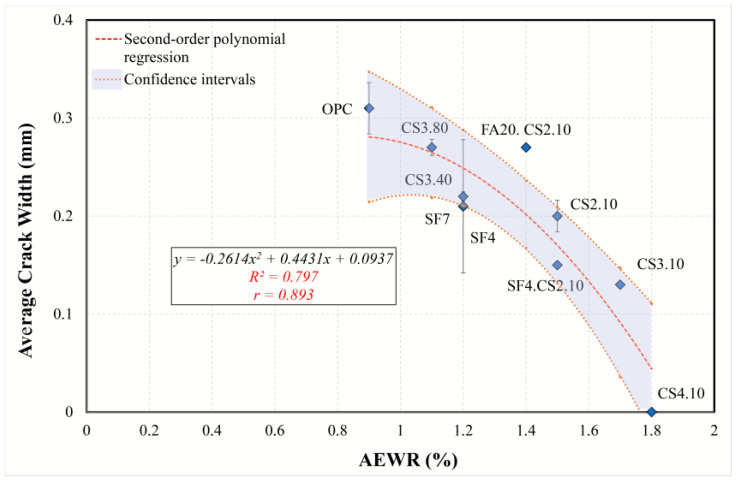
Second-order polynomial regression curve of AEWR versus average crack width.

**Figure 16:**
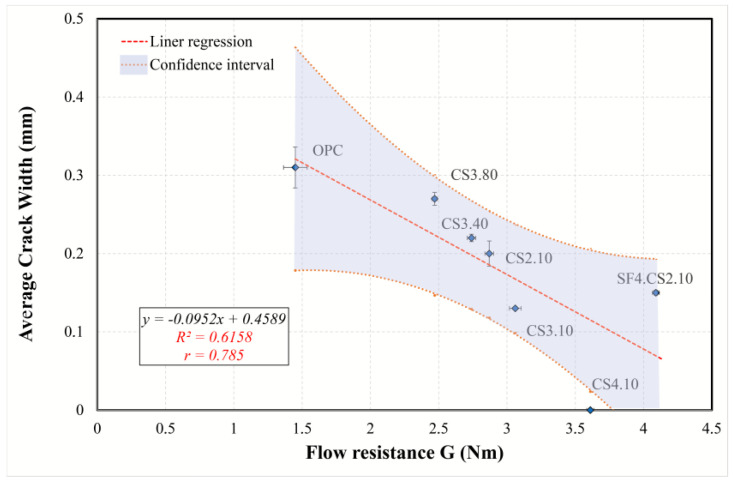
Linear regression curve explaining the relationship between flow resistance and average crack width.

**Figure 17:**
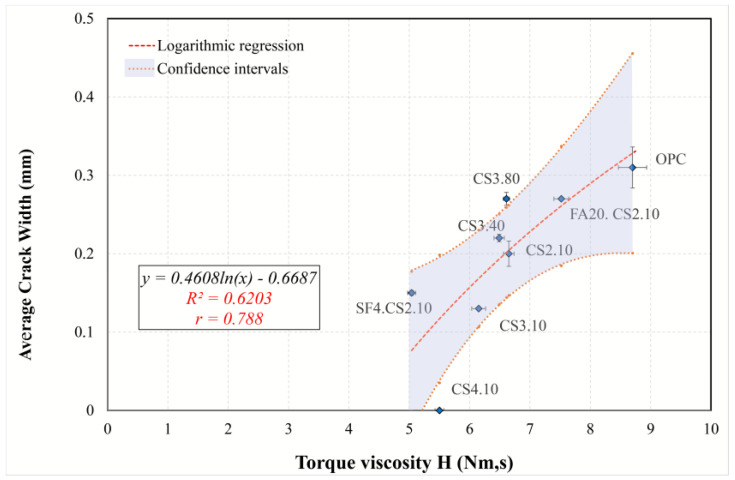
Logarithmic regression curve explaining the relationship between torque viscosity and average crack width.

**Figure 18:**
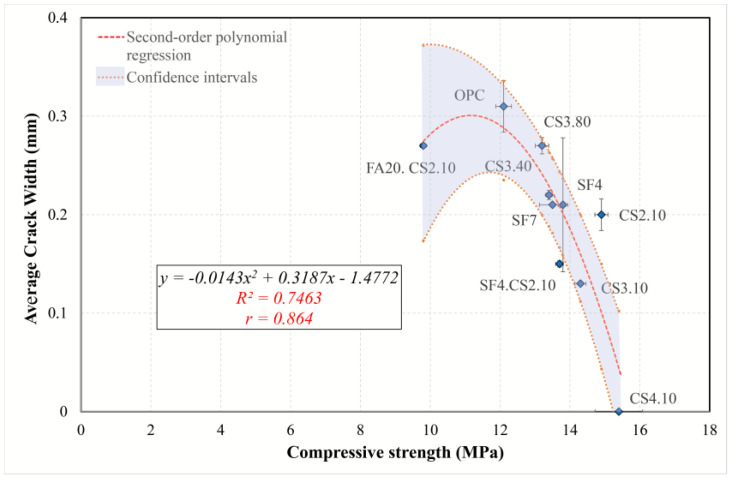
Second-order polynomial regression curve of compressive strength versus average crack width.

To gain a better understanding of the effect of different predictors on cracking, the simple regression models were examined and interpreted, and the most appropriate model was found. The regression analysis and information criteria are summarized in Table 7. Because the dependent variable (average crack width) contains a zero value (CS4.10), the application of logarithmic transformation is impossible (power, exponential, and growth models). Therefore, only linear, logarithmic, and polynomial second- and third-order models were considered. Although cubic regression has high determination coefficients and low *AIC* and *BIC* criteria for all variables, its graphs are parabolic or sinusoidal, which reduces its explanatory and interpretation abilities. Based on the analysis in Section 3.5.1, the following models can be identified:

- The AEWR model was analyzed using all mixtures, with the results indicating that all models are statistically suitable. However, the model best suited for this purpose was determined to be the quadratic model, as it showed the highest *R*^2^*adj* (0.739) and the lowest value of the information criteria. It was found that, according to the regression equation in Figure 15, an increase in the content of a water-reducing admixture by one unit (from 1 to 2%) results in a decrease in the average crack width by 0.341 mm (24%). Therefore, the water reducer can minimize the effects of plastic cracking for all types of mixtures.
- A linear model (Figure 16) was determined to be the best suited to describe the regression involving the flow resistance with only the mixtures containing colloidal silica (optimal regression statistics). The model shows that with an increase in *G* by 1 nm, the average crack width decreases by 0.09 mm (30%). Therefore, in the case of mixtures containing colloidal silica, a high value of the yield stress is associated with a reduction in plastic cracking.
- A simple model (Figure 17), including plastic viscosity, was interpreted by using logarithmic regression because second- and third-order polynomials are not significant, while a linear one has a smaller *R*^2^*adj*. This model was identified based on mixtures containing ultrafine fly ash and colloidal silica. Based on the analysis, the torque viscosity can be used to predict crack formation, and when it increases by 10%, the crack width increases by 0.044 mm.
- The model using compressive strength can characterize all mixtures (Figure 18). For this simple model, quadratic regression is best suited. Considering the curve, with an increase in strength from 12 to 15 MPa, the crack width decreases by 0.202 mm (70%). Consequently, it can be concluded that the compressive strength can perfectly explain the development of plastic cracking in the semi-plastic phase of all mixtures.

Considering the statistical analysis, it can be concluded that plastic cracking is influenced by all explanatory variables. However, after conducting additional analysis of multiple regression (Model IV), including AEWR, compressive strength, *G*, and *H* for all mixtures, the following results were obtained. The adjusted determination coefficient showed a strongly dependent relationship between plastic cracking resistance and these variables (*R*^2^*adj* = 0.783) and at a significance level of 1%. Separate model variables were not significant (owing to multicollinearity), indicating the complex nature of all the above predictors (the nonlinear effect of various variables on plastic cracking) while showing that there is a relationship between the yield stress and cracking for colloidal silicate and fly ash mixtures. Furthermore, because *G* and *H* are factors that influence the plastic phase, shear strength development in semi-plastic can reduce crack width (hold cracks). In other words, the additional presence of AEWR and strength development resulted in smaller crack widths in mixtures.

## 4. Discussion of the Results

Bleeding, evaporation, settlement, shrinkage, and capillary pressure build-up influence the rate and extent of plastic shrinkage cracking [74,75]. Further, the development of values of tensile stresses caused by surface tension forces occurs before the initial setting time and progresses up to the final setting time [3,4]. However, early strength development can withstand shear stresses and provide adequate stability. Therefore, plastic cracking is dependent on the influence of a set of factors, as shown in the statistical analysis (Section 3.5). Moreover, the characteristics of mixtures increase the risk of plastic cracking. For instance, an aggregate with a small maximum size and a low water–cement ratio can achieve the required physical and mechanical characteristics of shotcrete [76]. However, diverse admixtures such as accelerators, silica fume, and fly ash used to ensure optimal pumpability and shootability can also impair plastic cracking resistance [57,58,69,77]. Further, spraying can result in the formation of a rigid, compacted structure that is more susceptible to cracking [3,78]. This stiffness hinders the stage of bleeding, thereby increasing the influence of the evaporation process during the period when the mixture does not possess sufficient strength. 

The application of various fine particle-size cementitious materials results in an increase in the likelihood of cracking owing to a decrease in capillary pore size and an increase in pressure build-up (an increase in the number of menisci) [18]. Sirajuddin and Gettu also argued that high replacement of mineral admixtures can increase the intensity of plastic cracking caused by delaying setting and early strength [30]. However, the physical level of mineral admixtures (ability to absorb free water) also contributes to an increased risk of plastic cracking (by reducing bleeding) [79,80]. Furthermore, based on previous studies, it can be concluded that the specific surface area of mineral particles, which determines their fineness, influences the processes causing plastic cracking. In addition, the size and shape of the particles and the physical properties of the admixtures also affect the concrete rheology.

Papo et al. argued that the paste viscosity can be increased with high water adsorption and high interaction between particles caused by the high specific surface area of the particles [81]. In other words, a high specific surface area results in an increase in the attraction forces between the particles of the cementitious material and adsorbs more water molecules, thereby resulting in a high mixture viscosity. Moreover, Bentz et al. argued that the yield stress depends on the density of the cementitious particles, while the viscosity is influenced by the total particle surface area or total particle density [82]. Consequently, it can be concluded that the mineral admixture and cement rheology are influenced by the filler effect (i.e., an increase in the framework density) and the absorption effect of water molecules (i.e., unbound water absorption). Therefore, rheological properties and plastic shrinkage cracking exhibit a relationship.

In this study, the effect of colloidal silica application, as evident from the discussion in Section 3.1 (workability) and Section 3.4 (rheology), was examined. The decrease in workability may be because of the water absorption effect, while the increase and decrease in flow resistance and viscosity, respectively, are caused by the filler effect of particles because nanoparticles can fill interstitial spaces inside the framework of cement particles, thereby increasing its density and displacing water [38]. Moreover, the colloidal silica particles reduce the number of micropores in the mixture (filler effect) [38], thereby increasing the risk of capillary pressure build-up. In addition, the silica particles accelerate the hydration reaction owing to the larger formation of C-S-H gels and, subsequently, the more compact accumulation of these gels and a smaller resulting nanopore number [38,61,62], which affects the early strength development and crack resistance [83], as discussed in Section 3.3. These effects cause a moderate correlation between compressive strength and rheological parameters, and consequently, the resulting crack width decreases (Section 3.2). Therefore, an increase in the yield stress and a decrease in plastic viscosity are indicators of the plastic cracking resistance of mixtures containing colloidal silica.

Meanwhile, the particle fineness of ultrafine fly ash, coupled with its effect on the total binder powder granular composition, resulted in an increase in the yield stress, and further, owing to the absorption of water, an increase in viscosity occurred (Section 3.4). Study results [35] are consistent with this outcome, that is, fly ash particles increase the packing density. Simultaneously, many studies have indicated that a decrease in the cracking resistance of mixtures with fly ash addition [28,29,30] was also associated with water absorption. In this study, fly ash was combined with colloidal silica, and thus, an insignificant reduction in crack width was observed compared to OPC in another study [66]. However, coupled with the multiple regression Model IV, it is demonstrated that an inverse relationship exists between yield stress and plastic cracking. It may be that, as Kalovole highlighted, high-yield stress causes the capillary pressure and shrinkage to increase, and therefore, for a mixture with fly ash, a high *G* value is a factor of a decrease in cracking resistance [39]. This argument is consistent with the study [60], which states that the addition of small particle-size fillers increases capillary pressure. However, as with colloidal silica, a high torque viscosity is associated with a high plastic cracking risk (based on Model II regression) owing to water absorption and the resulting capillary pressure increase. In addition, the low compressive strength (Section 3.3) is caused by the retardation of the hydration reaction [65], which delays the initial and final setting times and early strength growth. Therefore, the correlation and regression analyses showed a good relationship between plastic cracking and compressive strength.

The silica fume used in this study showed a reduction in cracking compared to conventional Portland cement. Based on Model I, no relationship could be determined between rheological properties and plastic cracking for variables with silica fume, and only a relationship with compressive strength and water reducer content was found. This is confirmed by the fact that when the SF content changed from 4 to 7%, the crack width remained the same (Section 3.2), with an increase in the yield stress and a decrease in viscosity. However, when compared with OPC, an early strength development (24 h) was observed, which was the same for both series (Section 3.3), suggesting that the other factors were dominated by the strength development. In addition, an increase in the early tensile strength of the SF mixture at high capillary pressure was asserted [59]. Therefore, it is possible to conclude, according to Kalovole [39], that the high-yield stress of silica fume mixtures increases the capillary pressure, and hence, the risk of plastic cracking is increased by the high flow resistance. However, the acceleration of the hydration effect by silica fume [63,64] and the addition of a water reducer stopped the plastic cracking development.

**Figure 19:**
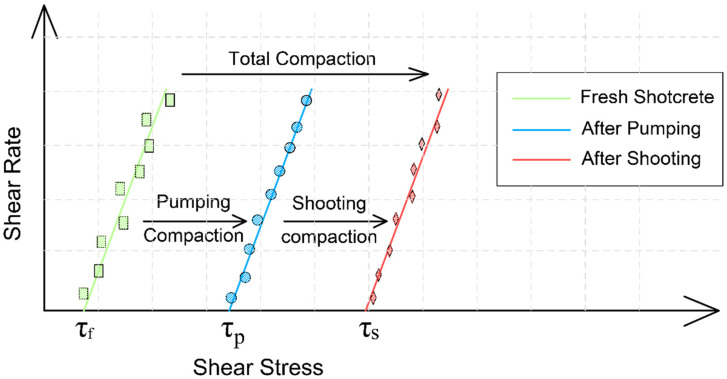
Shotcrete yield stress development as a result of spraying and pumpability compaction. Figure adapted from Beupre’s doctoral thesis [69].

In addition, the features of the shotcrete technology must be considered. Wet-mixed shotcrete consists of three main phases: mixture production, mixture pumping through hoses, and shooting through a nozzle. During the pumping process, the mixture is densified under pressure; moreover, the spraying action, that is, contact with the surface, causes the occurrence of the compaction process [69,84,85]. This effect is illustrated in Figure 19, which shows the change in the flow curve points depending on the pumping and spraying operations performed. Consequently, the yield stress increases, thereby increasing the stiffness, which hinders bleeding and, in accordance with the arguments (Kalovole), causes an increase in capillary pressure. Consequently, plastic cracking increases as well. However, controlling the change in yield stress with the ICAR rheometer is a challenge, as it is designed for flowable mixtures. Simultaneously, this is an important indicator for shotcrete, and an increase in the yield stress is indicated by a decrease in the air content after spraying (Section 3.1). Therefore, in the future, it is necessary to allocate time to studying this issue.

The formation of plastic shrinkage cracks is highly complex, and it can be attributed to several factors, including construction technology, environmental factors, mixture properties, and the material composition itself [86]. The supplementary cementitious materials used in this study can generally improve mechanical properties (for instance, CS and SF) or retard them (UFFA). Meanwhile, these admixtures can increase yield stress and viscosity, or vice versa, thereby affecting workability. All of these result in variation, which ultimately affects plastic cracking in terms of strength and the mechanics of fresh properties, that is, *G* and *H* are factors that influence the plastic phase, while shear strength development in semi-plastic can reduce crack width (hold cracks). Mixture compositions ultimately create our resulting properties; composition modifications also result in changes in the final properties, i.e., they are initially interconnected. Based on this concept, shrinkage cracking can be predicted and controlled, thereby satisfying the requirements for shotcrete durability.

## 5. Conclusions

The application of shotcrete is related to several deteriorating processes that start its formation in the early stages of the concrete’s life. Plastic shrinkage cracking is an illustration of these issues, which greatly affects the structure’s longevity. Plastic cracking kinetics is closely related to mineral admixture characteristics such as fineness and chemical composition. However, the fine particles lead to faster hydration and result in the acceleration of the onset of the initial and final setting times. The fine particles fill the pores, which increases the number of menisci formed and, consequently, increases the capillary pressure. Further, the high specific surface area of supplementary binders results in an increase in the interaction forces between the particles and free water demand and thus leads to the formation of a more compact framework structure, affecting rheology. In this study, the relationships between plastic shrinkage cracking and physicomechanical/rheological characteristics were analyzed, and the following findings were distinguished:

- The ultimate compressive strength development at an early age, determined by the hydration process acceleration, and the water-reducer content, determined by the capillary pressure reduction, affect all the mixtures considered in this study. In the case of 4 and 7% silica fume, these processes were observed to be the most prevalent; therefore, cracking was formed to a lesser extent compared to OPC. The high-yield stress of these mixtures demonstrates an increased capillary pressure owing to their high capacity to absorb free water and reduce capillary pore size.
- The crack width of the colloidal silica series (10 nm) was influenced by rheological parameters and early strength characteristics, whereas high flow resistance and low torque viscosity were found to be factors of reduced plastic shrinkage cracking. Moreover, this was also found to be true for solutions containing larger colloidal silica particle dimensions (40 and 80 nm), so their contribution to cracking reduction was less pronounced.
- For a mixture containing ultrafine fly ash, a high value of yield stress and plastic viscosity are attributed to capillary pressure development, coupled with the deceleration of hydration, resulting in lower cracking resistance. However, colloidal silica (2%) can stabilize FA (20%), and their combined application can counteract this degradation process.
- As a mixture containing colloidal silica and silica fume is similar in its properties to a simple colloidal silicate series, the smallest crack observed is because of high flow resistance and low torque viscosity, as well as the presence of AEWR and strength development. In addition, the combination of 2% CS and 4% SF showed a greater effect on the average crack width of the specimen.
- The statistical analysis showed that all influencing factors are interconnected in a complex manner, jointly affecting plastic cracking, that is, *G* and *H* are factors that influence the plastic phase, and shear strength development in a semi-plastic can control crack development. In addition, an increase in yield stress after spraying and the consequent reduction in bleeding can lead to an increase in capillary pressure, which can cause risks associated with shotcrete technology.

This study is expected to contribute to the further investigation of the concrete rheology influence on plastic shrinkage cracking. However, it should be noted that this relationship only applies to the admixtures (their content) used in this study. 

## Figures and Tables

**Table 1:** Characteristics of the materials and admixtures used.

Physicochemical Properties of Cement Materials								
Type	Chemical Composition (%)						Fineness (cm^2^/g)	Specific Gravity
	SiO_2_	Al_2_O_3_	Fe_2_O_3_	CaO	MgO	SO_3_		
Cement	20.8	6.3	3.2	61.2	3.3	2.3	3300	3.15
SF	97.66	0.13	0.20	0.11	0.11	-	220,000	2.22
UFFA	49.4	27.5	8.43	-	1.62	1.12	6934	2.58
Physicochemical properties of colloidal silica								
Average particle diameter (nm)		pH	Density (g/cm^3^)		Viscosity (cps)		Na_2_O (%)	SiO_2_ (%)
10–20		10.0	1.28		50		0.6	40
40–50		10.0	1.28		30		0.5	40
80–90		10.0	1.28		30		0.5	40
Physical properties of AEWR agent								
Density (g/cm^3^)	Alkali Amount (kg/m^3^)		Water Reduction rate (%)				Bleeding Ratio (%)	Application
1.05	0.01		22				48	Binder weight × 0.5∼2.5%

**Table 2:** Concrete mixture compositions.

Type	G_max_ (mm)	W/B	S/a (%)	Unit Weight (kg/m^3^)							
				Water	Cement	Sand	Gravel	Silica Fume	Fly Ash	Colloidal Silica	AEWR
OPC	10	0.4	75	184	460	1218	417				4.14 (0.9%)
CS2.10	10	0.4	75	179	460	1222	418			9.2	6.90 (1.5%)
CS3.10	10	0.4	75	176	460	1224	419			13.8	7.80 (1.7%)
CS4.10	10	0.4	75	173	460	1227	420			18.4	8.28 (1.8%)
CS3.40	10	0.4	75	176	460	1224	419			13.8	5.52 (1.2%)
CS3.80	10	0.4	75	176	460	1224	419			13.8	5.06 (1.1%)
SF4	10	0.4	75	184	442	1213	415	18			5.52 (1.2%)
SF7	10	0.4	75	184	428	1210	414	32			5.52 (1.2%)
SF4. CS2.10	10	0.4	75	179	442	1217	416	18		13.8	6.90 (1.5%)
FA20. CS2.10	10	0.4	75	179	368	1202	412		92	13.8	6.44 (1.4%)

**Table 3:** The experiment factors and parameters.

Run	Colloidal Silica (%)	Particle Size (nm)	Silica Fume (%)	Ultrafine Fly Ash (%)	AEWR (%)
1	-	-	-	-	0.9
2	2	10	-	-	1.5
3	3	10	-	-	1.7
4	4	10	-	-	1.8
5	3	40	-	-	1.2
6	3	80	-	-	1.1
7	-	-	4	-	1.2
8	-	-	7	-	1.2
9	2	10	4	-	1.5
10	2	10	-	20	1.4

**Table 4:** Rheological properties of shotcrete containing different supplementary cementitious materials.

Mixtures	Intercept	Slope	*R* ^2^	Standard Error	*G* (Nm)	*H* (Nm,s)
OPC	0.163	0.114	0.994	0.015	1.45	8.7
CS2.10	0.430	0.150	0.998	0.007	2.87	6.65
CS3.10	0.496	0.162	0.997	0.010	3.06	6.15
CS4.10	0.653	0.181	0.998	0.007	3.61	5.5
CS3.40	0.422	0.154	0.998	0.007	2.74	6.49
CS3.80	0.374	0.151	0.995	0.004	2.47	6.61
SF4	0.492	0.179	0.995	0.013	2.75	5.54
SF7	0.764	0.219	0.994	0.015	3.48	4.56
SF4.CS2.10	0.810	0.198	0.998	0.007	4.09	5.04
FA20. CS2.10	0.696	0.133	0.997	0.009	5.25	7.52

**Table 5:** Grouped variables to study their contribution to the development of average crack width.

Explanatory Variables							Dependent Variable
Mix Composition	Plastic Phase				Semi-Plastic Phase		
	Air Content		Rheological Parameters		Strength Characteristics		
AEWR (%)	Before shooting (%)	After Shooting (%)	*G* (Nm)	*H* (Nm.s)	Compressive Strength (MPa)	Flexural Strength (MPa)	Average Crack Width (mm)

**Table 6:** Multiple regression model analysis.

Model		Model I	Model II	Model III				
				All Explanatory Variables	*G* and Compressive Strength	*H* and Compressive Strength	*G* and AEWR	*H* and AEWR
Regression equation		Y = 0.78 −0.213x_1_ −0.022x_2_	Y = 0.638 −0.203x_1_ +0.011x_2_ −0.018x_3_	Y = 0.598 −0.254x_1_ −0.012x_2_ +0.001x_3_ −0.003x_4_	Y = 1.068 −0.046x_1_ −0.054x_2_	Y = 0.785 +0.029x_1_ −0.057x_2_	Y = 0.585 −0.013x_1_ −0.263x_2_	Y = 0.497 +0.009x_1_ −0.266x_2_
Regression Statistics	R Square	0.863	0.877	0.870	0.800	0.785	0.870	0.870
	Adjusted R Square	0.824	0.785	0.610	0.700	0.677	0.805	0.805
ANOVA test	Significance	0.001	0.027	0.243 *	0.040	0.046	0.017	0.017
*p*-value	AEWR (%)	0.004	0.072 *	0.418 *	-	-	0.049	0.040
	G (Nm)	-	-	0.948 *	0.283 *	-	0.742 *	-
	H (Nm,s)	-	0.669 *	0.993 *	-	0.344 *	-	0.750 *
	Compressive strength (MPa)	0.044	0.241 *	0.970 *	0.127 *	0.119 *	-	-
Information criteria	AIC	−6.554	−6.093	−5.898	−5.753	−5.680	−6.183	−6.181
	BIC	−6.493	−6.064	−5.922	−5.769	−5.695	−6.199	−6.197

* Significance level below 0.05.

**Table 7:** Appropriate model selection.

Regression Equations	Regression Statistics	Crack Width Factors				Regression Equations	Regression Statistics	Crack Width factors			
		AWER	*G*	*H*	Compressive Strength			AWER	*G*	*H*	Compressive Strength
Linear y = a + bx	*R* ^2^	0.745	0.616 ^#^	0.613	0.506	Quadratic y = a + b_1_x + b_2_x^2^	*R* ^2^	0.797 ^#^	0.623	0.626	0.746 ^#^
	*R* ^2^ *adj*	0.713	0.539 ^#^	0.548	0.444		*R* ^2^ *adj*	0.739 ^#^	0.434	0.477	0.674 ^#^
	*p*	0.001	0.037 ^#^	0.022	0.021		*p*	0.004 ^#^	0.142 *	0.085 *	0.008 ^#^
	*AIC*	−6.132	−5.421 ^#^	−5.446	−5.470		*AIC*	−6.359 ^#^	−5.440	−5.482	−6.136 ^#^
	*BIC*	−6.101	−5.428 ^#^	−5.436	−5.439		*BIC*	−6.329 ^#^	−5.447	−5.472	−6.106 ^#^
Logarithmic y = b + alnx	*R* ^2^	0.702	0.602	0.620 ^#^	0.452	Cubic y = a + b_1_x^2^ + b_2_x^3^	*R* ^2^	0.809	0.956	0.629	0.745
	*R* ^2^ *adj*	0.665	0.523	0.557 ^#^	0.383		*R* ^2^ *adj*	0.755	0.912	0.481	0.672
	*p*	0.002	0.040	0.020 ^#^	0.033		*p*	0.003	0.016	0.084 *	0.008
	*AIC*	−5.976	−5.387	−5.466 ^#^	−5.365		*AIC*	−6.422	−7.584	−5.489	−6.131
	*BIC*	−5.946	−5.394	−5.456 ^#^	−5.335		*BIC*	−6.392	−7.591	−5.479	−6.100

* Significance level below 0.05. ^#^ Optimal model.

## Data Availability

Raw data were generated at Kangwon National University. Derived data supporting the findings of this study are available from the corresponding author on request.

## Abbreviations

AC: air content after spraying; AEWR: air-entraining water-reducing agent; BC: air content before spraying; *CRR*: crack reduction ratio; CS: colloidal silica; FA: fly ash; OPC: ordinary Portland cement; RH: relative humidity; S/a: sand/aggregate ratio; SF: silica fume; SCM: supplementary cementitious materials; UFFA: ultrafine fly ash. *AIC*: Akaike penalized-likelihood criteria; *BIC*: Bayesian penalized-likelihood criteria; *b*: width of the failed cross-section (mm); *d*: diameter of specimen (mm); *f_b_*: flexural strength (MPa); *f_c_:* compressive strength (MPa); *G*: flow resistance (Nm); G_max_: maximum grain size (mm); *H*: torque viscosity (Nm.s); *l*: span length (mm); *P*: maximum obtained load (N); *r*: Pearson correlation coefficient; *R*^2^: determination coefficient; *R*^2^*adj*: adjusted determination coefficient.
